# The (Un)fairness of Vaccination Freeriding

**DOI:** 10.1093/phe/phac028

**Published:** 2022-11-14

**Authors:** Marcel Verweij

**Affiliations:** Wageningen University, The Netherlands

## Abstract

For contagious diseases like measles a successful immunization program can result in herd protection. Small outbreaks may still occur but fade out soon, because the possibilities for the pathogen to spread in the ‘herd’ are very small. This implies that people who refuse to participate in such a program will still benefit from the protection it offers, but they don’t do their part in maintaining protection. Isn’t that a case of freeriding—and isn’t that unfair towards all the people who do collaborate? If so, that might be considered an additional ground for making vaccination mandatory or compulsory.

In this paper I argue that vaccination refusal can be considered as freeriding, but that this might not be unfair. The public good of herd protection is a peculiar public good because it supervenes on private benefits that are enjoyed by all who do opt for vaccination. For vaccinated individuals, the additional benefit of herd protection comes about, as it were, for free, and hence they can’t complain that others benefit without sharing in the burdens. There are however still other grounds for making vaccination compulsory or at least for seeing refusal as a morally wrong choice.

## Herd Immunity, Freeriding and Compulsory Vaccination

A major factor in the success of many collective immunization programs is that they can result in herd protection. If a sufficiently large part of the population has become immune, it will be impossible for a contagious disease to spread from one infected person to many others, and, although small outbreaks may still be possible, these will fade out rapidly. In some cases it may even be possible to eliminate or eradicate the disease altogether. Such a group level protection is a clear example of a public good. The benefits are open to all members of the population: everyone benefits from the fact that no large, possibly disruptive outbreaks will occur. Moreover, herd immunity will also protect individuals who have not or cannot be immunized themselves. For example, persons who cannot be vaccinated for medical reasons, children who are too young to be vaccinated, and individuals that respond insufficiently to the vaccine, due to another illness, or to immunosuppressive therapy. And, interestingly, it also protects persons who forego, postpone or actively refuse vaccination. In this way, collective vaccination programs that result in herd immunity against certain infectious disease have a feature that most public goods have: the benefits are open to everyone, irrespective of whether or not people have contributed, and this creates the possibility of freeriding. People can opt out of vaccination programs and still be protected (themselves or their children) by the fact that the collective program has resulted in group-level protection, so they will run little risk to be infected and get ill.

Freeriding is often considered to be unfair, and a relevant question for vaccination policies is whether this offers a prima facie ground for governments to set limits to the right of citizens to refuse medical treatment and to decide not to participate in a collective vaccination scheme. For example, fairness is an important element of Alberto Giubilini’s (2019) argument for making immunization compulsory. In this paper I will argue that vaccination refusal indeed often does amount to freeriding, but that this cannot be considered as unfair.

For that matter, I do assume that under certain conditions mandatory or compulsory vaccination can be justified in a liberal democracy, but this justification would be framed in terms of John Stuart Mill’s principle: to prevent harm to others (Mill, 1859).^1^ The question for now is whether the unfairness of vaccination free riding offers an additional argument for compulsion. Such additional ground would be welcome for proponents of coercive vaccination policies, because the application of the harm principle to immunization faces a weakness, and the additional fairness argument would specifically help to repair that. The limitation of the harm principle argument is that, in a context where vaccine coverage is already more than sufficient for herd immunity, opting out of the scheme appears a rather innocent choice that does not impose risks on others. If group immunity has been secured, everyone is protected, and incidental outbreaks will fade out immediately. If a parent would then decide to forego vaccination for her child, it might make no significant difference to others—not even to the child itself. Yet if such a choice can be considered as freeriding upon the cooperative efforts of other citizens, and if this is unfair, there is a strong ground for coercive regulation after all—even if the participation amounted to almost 100%. The argument would be analogous to how it is fully justified to compel citizens to pay their taxes even if the government budget has a surplus: the issue is not that some individual’s contribution is necessary for government policies. The argument for compulsion is that all citizens must be treated equitably, and that the burdens of taxes should be distributed fairly. In normal circumstances (assuming a democratically legitimate tax regime) refusing to pay taxes is clearly unfair. But is vaccine freeriding also unfair?

## Vaccination refusal as freeriding

Let us unpack the argument and discuss first whether vaccination refusal amounts to free riding before continuing to explore the issue of fairness. Although it is often difficult to disentangle descriptive and normative elements in such a thick concept, Garett Cullity (2013) does a good job:

“Free riding,” used as a descriptive term, refers to taking a jointly produced benefit without contributing towards its production. Used as a term of criticism, it refers to the wrongful failure to contribute towards the joint production of benefits that one receives.

Can vaccination refusal be considered freeriding in the descriptive sense—as the taking of a jointly produced benefit without contributing towards its production? That will depend on how we understand ‘taking’—which is also not without normative or pejorative dimensions. There is at least one type of vaccine refusal that clearly does *not* satisfy this definition. Parents who endorse Rudolph Steiner’s anthroposophist world view refuse vaccination because they see ‘childhood illness’ like measles or pertussis as enabling important steps in childhood development. But if these groups reject vaccination because they see the illness as beneficial for their children, they will also reject the idea that avoiding the disease would count as a benefit. They prefer seeing their child getting ill with measles—of course with the expectation or hope that the child will recover—rather than avoiding the infection. It is not uncommon for parents in these circles, when they see their child having symptoms of measles, to organize a ‘measles party’ and invite other children, to enable them to be exposed as well. It is clear therefore that these parents do not ‘take the benefits’ of herd protection, and consequently we cannot reasonably call their refusal an instance of freeriding. Anthroposophist worldviews are prominent in some regions (e.g. Switzerland and southern parts of Germany) and a common factor in measles outbreaks in those regions, but these groups only represent a small subset of all vaccine refusers.

At the other extreme of the spectrum, cases of *intentional vaccination freeriding* (deliberate foregoing of vaccination because one prefers to benefit from the vaccinations of others), are also not very common. The fact that they probably do exist can be illustrated with Robert Sears, a USA anti-vaccination celebrity also known as ‘Dr Bob,’ who is blatantly honest in his advice to non-vaccinating parents “I also warn them not to share their fears with their neighbors, because if too many people avoid the MMR vaccine, we’ll likely see the disease increase significantly” (Sears 2007, 96–97, as quoted in Navin, 2016, p. 143). This advice, deleted in the second edition of his book (Sears, 2011), clearly reflects a deliberate intention to ‘take the benefits’ of the cooperative endeavor and thus to freeride on other people’s willingness to vaccinate.

Most vaccination refusers however are neither as clearly a freerider as Dr Bob, nor do they embrace the other extreme position that it is good for a child to be infected with measles. They forgo vaccination because they consider the risks of vaccination as outweighing any benefits, or they do not trust vaccinations (or the government and the pharmaceutical industry) altogether.^2^Bradley and Navin (2021) therefore argue that this range of choices does not amount to freeriding at all: it does not involve an intentional choice to take the benefit without reciprocating. This conclusion is questionable. Presumably all these citizens want to avoid illness for themselves or for their child, and in that sense they do clearly take benefits from herd immunity—that is, they ‘take’ the absence of illness or the remoteness of risk as something good. Some of them might acknowledge the fact that infection risks are low is the outcome of a high immunization rate. Most of them probably do not bother about the fact that they and their children are protected by herd immunity. They simply see the (actually very small) risk of a measles or pertussis infection as acceptable and can thus reasonably opt to forego vaccinations for the reasons they give: the alleged side effects of vaccines or distrust in government and pharmaceuticals. But of course, the acceptable risk is a result of successful collective vaccination program to which a large majority of citizens have contributed by participating. In this way, they do take a ‘free ride’ even though few of them will perceive it in that way: their assessment of the risks and benefits of immunization takes the low incidence of disease—caused by high immunization rates—as given and they opt out on the basis of that assessment. The leaves us with the question: is such freeriding unfair?

## How Vaccination Refusal May Seem Unfair

Before analyzing this stance about the unfairness of vaccination freeriding, let us briefly take a closer look at the concept of fairness itself and try to grasp what makes unfair practices wrong. This is not an easy task, because ‘fairness’ is such a basic and intuitively appealing idea that it is hard to pin it down in straightforward terms. Arguably John Rawls has made most work of explaining fairness, as it is at the heart of his theory of justice, but even Rawls seems unable to offer a precise definition. This is probably the best we can get:

“The question of fairness arises when free persons, who have no authority over one another, are engaging in a joint activity and amongst themselves settling or acknowledging the rules which define it, and which determine the respective shares in its benefits and burdens. A practice will strike the parties as fair if none feels that, by participating in it, they or any of the others are taken advantage of, or forced to give in to claims which they do not regard as legitimate” (Rawls, 1958, p. 178).

Fairness only makes sense in a context in which free and reasonable people acknowledge certain rules as legitimately guiding how they should relate to one another. A fair distribution of burdens and benefits of social cooperation is then one that is determined by general principles that all can agree with. If certain beneficial goods can only be made available to anyone if (almost) all contribute to its creation—as in a public good—and if such contribution comes with not-insignificant costs, then it is reasonable for people to reject free-riding: an individual deliberately reaping the benefits without contributing and thus to take advantage of those who do contribute.

The non-collaborator is using the cooperative and burdensome contribution of others as a means to be better off himself, only ‘participating’ in the collective endeavor as it comes to distribution of benefits, and not where it involves the contributions. This shows a lack of respect for others as free and equal individuals, and also for the importance of having mutual beneficial practices and rules. Moreover, by reaping, hence accepting, those benefits, others can reasonably claim that he voluntarily commits himself to the societal scheme and thereby to an obligation to do his fair share. It is like freely deciding to enter a particular societal practice or game: this commits one to follow the rules of the game as well. In Rawls’s view on public goods, accepting the benefits is one of the conditions for seeing cooperative action as something that is morally required as a matter of fairness (Rawls, 1971, pp. 111–112).

Whether or not *accepting* the benefits is a necessary condition for seeing freeriding as unfair, has been a matter of philosophical debate.^3^ Yet whether or not one follows Rawls’s more restricted account of unfairness, it seems clear that Robert Sears’s advice as quoted above, amounts to unfair freeriding. The same applies—though less visible—to vaccine refusers who see the risk of immunization as outweighing the remote benefits. As I argued in response to Bradley and Navin, these parents can only consider the benefits as being remote given the fact that most people are immunized and thus have contributed to herd protection. In this way, refusers do assume the benefits of the public good. Mariëtte van den Hoven, therefore argues that all vaccination refusers who benefit from herd immunity, engage in unfair freeriding, simply because they choose not to do their fair share in the collective scheme (van den Hoven, 2013).

The unfairness of vaccination refusal is also central in Alberto Giubilini’s argument for compulsory immunization, although he does not focus on the aspect of freeriding. For Alberto Giubilini (2019), the starting point is that citizens *collectively* have an *obligation* to contribute to important public goods. This is an appealing idea in many collective action problems, and certainly for the current challenge of global warming. The harmful effects that climate change brings for current and future generation are enormous, and, given that global warming is clearly the result of the emissions that come with our modern way of living, humanity should take responsibility for preventing further emissions and mitigating the effects of climate change. It makes sense to see this responsibility as a collective obligation as we are creating the problem together, and also because individual attempts to mitigate climate change will be futile unless embedded in a concerted action together with many others.

In a similar vein, contagious diseases are spreading from human to human—which involves all of us as causal links in the spread of disease. Collective protection will require concerted efforts, and this can be seen as offering support to a collective responsibility to achieve herd immunity. The question is, however, how such joint obligation relates to duties for each individual citizen. Giubilini focuses on how the benefits and burdens of the collective obligation are distributed:

Thus, the collective obligation to realize herd immunity generates a certain amount of “burdens”: a certain number of individuals will have to be vaccinated. I call vaccination “a burden” in this context because some people are opposed to it and because vaccination does involve some small inconvenience (possible temporary pain of the injection, having to pay a visit to the doctor, potentially a financial cost, minor risk of some side effects, etc.). […] In any case, the relevant question, for our purposes, is the question as to how such burdens should be distributed among individuals who form the collective with the moral obligation to realize herd immunity. It is safe to assume that such burdens should be distributed fairly, to the extent that we think that fairness is an important value that needs to be taken into account when distributing any kind of burden involved in the realization of important public goods. Thus, fairness demands that each individual does whatever she reasonably can in order to contribute to the fulfilment of the collective or shared obligation, regardless of the actual impact any individual action would have on the realization of the collective outcome (Giubilini, 2019, pp. 50–51).

In other words, the principle of fairness requires that any individual—at least those who can contribute to herd immunity, and who can bear the (small) burdens—should take their fair share in fulfilment of the collective obligation. Of course, this involves only minor inconveniences and remote side effects, but still it makes sense to distribute burdens and benefits in an equitable way. If there are various ways to divide and distribute benefits and if the stakes of a collective project are very high, that is, if both the benefits but also the burdens are significant for most people involved, we might need to discuss in more detail what an equitable distribution involves. Should individuals who are worst off in some sense be exempted from sharing in the burdens? Or should those who contribute most to the collective risk (i.c. spread of infection) take a larger share? In vaccination programs there is only one way to contribute, to get a vaccination, and the burdens are rather mild. In this context, a simple understanding of fairness may be sufficient: enjoying the benefits of a collective project but refusing to contribute and share in the burdens of it, is not fair. So, if vaccination refusal involves an inequitable distribution of burdens and benefits, and refusers are deliberately choosing to refrain from sharing in the burdens yet they do benefit from the collective schemes, this looks like unfair freeriding—but is it really?

## The Peculiar Public Good of Herd Immunity: Freeriding Is Not Unfair

Central in our understanding of the wrongness of freeriding is that the distribution of benefits and burdens involved with the production of a public good, should be fair. This involves at least that benefits are open to all, and that the costs are shared equitably. In this section I will argue that the distribution of benefits and costs—and especially the costs—that come with maintaining herd protection, while some are taking a free rid, is *not* unfair. This implies that neither vaccination freeriding, nor immunization policies that allow room for freeriding, are unfair.

The ultimate reason why vaccination freeriding cannot be unfair, is that the public good of group-level protection *supervenes* on the private benefits of all who vaccinate. It only comes about as a collective result of the private goods that every vaccinating individual enjoys. In this it differs from many if not most other public goods. By definition, a normal public good can only come about if there is sufficient cooperative action (realized through government policy or more spontaneous collective efforts), and if the good comes about, then everyone benefits: it is non-excludable. If there is insufficient support, or if otherwise the collective effort is insufficient, the collective good will not be created, and no one will benefit. If a dike along the riverbanks has been realized for only 90% of the required length, then everyone will have wet feet, or worse, and the efforts of the contributors to build the dike have been in vain. Either the dike is strong enough, and it holds for everyone, or it is not, and then no-one is protected, and everyone’s contribution was useless. In other cases of suboptimal participation, people will still benefit to some extent, in relation to the proportion of citizens that were willing to cooperate. Take for example the case of collective efforts of fishermen to refrain from polluting a lake: if at least many are willing to cooperate, most of the pollution will be averted and everyone benefits at least a bit. But here, too: the benefits that each individual receives in return for the costs she makes will completely depend on the collaboration of others.

Interestingly, the public good of herd immunity comes about only via individuals who achieve personal immunity against infection, each for themselves or their own children. It is unlikely that many citizens opt for vaccination solely for the sake of contributing to herd immunity: most will seek vaccination to be protected themselves (Bradley and Navin 2021). This comes at some costs, inconveniences and even a very small risk of more severe side-effects such as fever convulsions, but it is reasonable for people to see their individual health benefits as by far outweighing the burdens. Now, if sufficient people have managed to receive protection for themselves or their children, then group protection arises as a ‘added benefit’. Whether or not this added group benefit is attained, every vaccinated individual will benefit from her own vaccination anyway—apart from a few individuals in whom the vaccine fails to be effective. If the number of people who participate is insufficient for group protection, then almost each vaccinated individual will still be protected and benefit; this is exactly happening in HPV vaccination campaigns that only target girls, and not boys.

Herd protection is an important public good that comes about as a result of many people successfully having secured protection for themselves and their children. *It is thus a public good that supervenes on private goods.* This is what makes herd immunity a peculiar public good, which differs in important respects from other public goods, such as the maintenance of dikes or the mitigation of climate change. For each and every individual vaccinated person, the attainment of herd protection offers only a minor, if not negligible added benefit, which is not to say that, for society at large it is insignificant. Herd immunity does protect all children too young to be vaccinated and other vulnerable groups such as frail elderly or immunocompromised patients. Vaccination refusers also clearly benefit from collective protection, while they do not contribute to the collective effort to achieve herd immunity—hence they do not share in the burdens.^4^ But are there any burdens at all for individuals who contribute to herd immunity? They achieve personal immunity for themselves or their children and thus are (individually!) protected against infectious diseases, and it is reasonable for them to consider this as outweighing possible minor burdens. Vaccination offers them individual protection, and if vaccine coverage is sufficient, herd immunity is an added benefit, which comes about, as it were, for free: at no additional cost for anyone. Therefore, those who vaccinate cannot complain that free riders unfairly reap the benefits without sharing in the burdens: from the perspective of vaccinating individuals, there are no burdens at all in producing herd protection. Herd immunity is a public good that supervenes on the private goods that vaccination citizens attain for their children or themselves; it is an ‘added benefit’ that puts no burdens on cooperating citizens. But if herd immunity constitutes a benefit for all at no extra cost or burden for contributors, then the distribution of burdens and benefits that results from the fact that some people take a free ride, cannot be unfair.

This line of reasoning might not work for all vaccination programs. For example, it might not apply to vaccination programs that target groups who can easily spread the infection but are not themselves vulnerable to the disease, to protect others who are vulnerable (Kraaijeveld, 2020). Think of proposals to offer influenza vaccination to all schoolchildren to primarily benefit the elderly (Savulescu et al, 2021). Or programs that encourage HPV vaccination among boys, primarily to protect girls against risks of cervical cancer at later age. It is not obvious that in such a case everyone who vaccinates will enjoy individual benefits that outweigh their burdens. Yet in such a case, it is also not clear that individuals who opt out, are themselves really benefiting from group-level protection, given that the risks or infection primarily hit other groups. Hence, their choice to opt out cannot be considered as freeriding. These cases therefore do not affect my analysis about freeriding.

## Conclusion

I have argued that herd immunity is a public good that supervenes on private goods that collaborators enjoy, and that therefore vaccination refusal does not result in an unfair distribution of burdens and benefits of collective vaccination.

This does not imply that vaccination freeriding is morally neutral: it is still wrong because it undermines herd protection and thus imposes a risk on others. Moreover, freeriding can be considered as morally objectionable as it shows a lack of solidarity with vulnerable persons who depend on herd protection. Even stronger: free riders occupy the ‘protective seat’ in the herd that was meant for the most vulnerable persons, and that can be rightly considered as morally repulsive.

The harm principle also still offers ground for vaccination policies that constrain individual freedom. Coercive programs can in principle be justified in a liberal democracy: vaccine refusal does undermine and obstruct the goal of achieving and maintaining herd immunity and that offers a principled ground for constraining the freedom of individuals to choose to opt out. To what extent coercive programs are necessary and proportionate in preventing harm, especially given the fact that no full compliance is necessary for achieving herd protection, is a different matter, which requires more in-depth analysis, taking many contextual factors into account. The idea that refusal also amounts to freeriding does however not offer an additional argument for coercion.

## Footnotes

^1^
The claim that mandatory policies can be ethically and legally justified is not uncontroversial, but certainly common among scholars in public health law and ethics (see for example Giubilini 2019; Flanigan 2014; Verweij 2011; Pierik & Verweij 2019; Dawson 2007; Caplan 2013).

^2^
Another group has religous objections against immunization: they see a choice for vaccination as a lack of trust in (or even a counteract to) Divine providence. Their motivation seems rather independent of the benefits of disease or immunity and hence may not be easily considered as a matter of freeriding.

^3^
See for example Cullity (1995) for an analysis of the unfairness of freeriding that does not require explicit acceptance of the benefits of a collective scheme.

^4^

Bradley and Navin (2021) argue that therefore people cannot both contribute to herd immunity and benefit from it: if they contribute, they will be immune themselves and herd immunity will not offer further health protection. Note however that the benefits of herd immunity go beyond health benefits: group-level protection will also prevent outbreaks that can disrupt social life, and this way, people who opt for vaccination do benefit from herd immunity after all.
